# Mesenchymal stem cells-derived extracellular vesicles ameliorate lupus nephritis by regulating T and B cell responses

**DOI:** 10.1186/s13287-024-03834-w

**Published:** 2024-07-18

**Authors:** Cuifang Li, Feifeng Wu, Jueyi Mao, Yang Wang, Junquan Zhu, Kimsor Hong, Haotian Xie, Xin Zhou, Jidong Tian, Chuan Wen

**Affiliations:** 1 Department of Pediatrics, The Second Xiangya Hospital of Central South University, Changsha, 410011 China; 2 Department of Gastroenterology, The Second Xiangya Hospital of Central South University, Changsha, 410011 China

**Keywords:** Lupus nephritis, Mesenchymal stem cells, Extracellular vesicles, T cell, B cell

## Abstract

**Background:**

Human umbilical cord mesenchymal stem cells-derived extracellular vesicles (hUCMSC-EVs) have potent immunomodulatory properties similar to parent cells. This study investigated the therapeutic effects and immunomodulatory mechanisms of hUCMSC-EVs in an experimental lupus nephritis model.

**Methods:**

The hUCMSC-EVs were isolated by using differential ultracentrifugation. In vivo, the therapeutic effects of hUCMSC-EVs in lupus-prone MRL/lpr mice were investigated, and the mechanisms of treatment were explored according to the abnormal T and B cell responses among both the spleen and kidney.

**Results:**

MRL/lpr mice treated with hUCMSC-EVs reduced proteinuria extent, serum creatinine and renal pathological damage; decreased splenic index and serum anti-dsDNA IgG level; and improved survival rate. hUCMSC-EVs lowered the percentage of T helper (Th)1 cells, double-negative T (DNT) cells, and plasma cells among splenocytes; inhibited the infiltration of Th17 cells but promoted regulatory T (Treg) cells in the kidney, followed by a reduction in pro-inflammatory cytokine levels(IFN-γ, IL-2, IL-6, IL-21, and IL-17 A). In addition, hUCMSC-EVs inhibited the activation of STAT3 and down-regulated IL-17 A protein levels in the kidney.

**Conclusion:**

The results of this study demonstrated that hUCMSC-EVs had therapeutic effects on experimental lupus nephritis (LN) by regulating Th1/Th17/Treg imbalance and inhibiting DNT and plasma cells. Additionally, hUCMSC-EVs inhibited Th17 cell differentiation in kidney by regulating the IL-6/STAT3/IL-17 signal pathway, which might be an important mechanism for alleviating renal injury. Taken together, we demonstrated that hUCMSC-EVs regulating T and B cell immune responses might represent a novel mechanism of hUCMSCs in treating LN, thus providing a new strategy for treating LN.

**Supplementary Information:**

The online version contains supplementary material available at 10.1186/s13287-024-03834-w.

## Introduction

Systemic lupus erythematosus (SLE) is a multisystem autoimmune disease commonly affecting the kidney [1]. Lupus nephritis (LN) is a significant risk factor for morbidity and mortality [1]. Traditional drugs, including glucocorticosteroids, immunosuppressants, and biologics, have not yet been able to address SLE recurrence well [2] and have significant adverse effects with long-term use [3]; hence, the development of highly effective therapies with few and mild side effects has become a clinical treatment challenge and attracted much attention.

Abnormal activation and imbalance of immune cells, such as T cells, B cells, macrophages, and dendritic cells, are important pathogenesis of SLE [4]. In recent years, human umbilical cord mesenchymal stem cells (hUCMSCs) have achieved remarkable efficacy in the clinical treatment of severe and refractory LN with their powerful immunomodulatory properties [5–7]. Studies reported that mesenchymal stem cells (MSCs) may correct the abnormal immune responses through secretory effects, such as the secretion of prostaglandin E2 (PGE_2_) [8], Indoleamine 2,3-Dioxygenase (IDO) [8], transforming growth factor (TGF) -β1 [8, 9], and nitric oxide (NO) [10]. However, the detailed secretion of MSCs other than the cytokines mentioned above is not known.

MSCs derived extracellular vesicles (MSC-EVs) are tiny vesicles with a bilayer lipid membrane structure. MSC-EVs contain biologically active molecules such as proteins, lipids, nucleic acids, and organelles (e.g., mitochondria) of MSCs origin. MSC-EVs transported bioactive cargo to various immune cells to regulate their number and function [11]. It has been reported that MSC-EVs showed similar therapeutic effects to their parent MSCs in some autoimmune diseases such as rheumatoid arthritis, type I diabetes, inflammatory bowel diseases, and multiple sclerosis [12]. To date, only two studies have investigated the therapeutic effects of MSC-EVs on LN, in which MSC-EVs reduced glomerular mesangial expansion, glomerular fibrosis, and immune complex deposition in the kidneys of lupus-prone MRL/lpr mice by modulating macrophages polarizing to an anti-inflammatory phenotype [13, 14]. However, these studies assessed the effects of MSC-EVs on LN mainly focus on renal pathology, and the specific mechanism still needs to be discovered. We previously identified that hUCMSC-EVs suppressed CD4^+^ T cells and regulated Th1 and Th17 cell differentiation in vitro [15]. Whereas T and B cell immune responses play an important role in the immune mechanism of SLE [4], it is unclear whether hUCMSC-EVs can ameliorate experimental LN by regulating T and B cell immune responses. Moreover, kidney-infiltrating T cells (KITs) are the most predominant type of kidney-infiltrating immune cells(KIIs) [16, 17], which directly affect kidney tissue and might contribute to one of the important determinants of LN [18]. However, little has been known about the immunomodulatory effects of MSCs or MSC-EVs on kidney-infiltrating immune cells since a much greater focus in murine and human lupus studies has been on the more readily accessible peripheral cell populations (e.g., spleen and peripheral blood cells).

In this study, we sought to explore the therapeutic effects of umbilical cord mesenchymal stem cells-derived extracellular vesicles (hUCMSC-EVs) on experiment LN and identify the immunomodulatory effects and mechanisms of hUCMSC-EVs on T and B cells among both splenocytes and KIIs *in vivo.*

## Materials and methods

### Animals

Eight-week-old female MRL/lpr mice and 8-week-old female C57BL/6 mice were purchased from Shanghai SLAC Laboratory Animal Co., Ltd (Shanghai, China) and Hunan SJA Laboratory Animal Co., Ltd (Hunan, China), respectively. The animals were maintained in a specific pathogen-free environment in the Animal Experimental Center of the Second Xiangya Hospital of Central South University. Experiments were carried out according to the National Institutes of Health Guide for Care and Use of Laboratory Animals and were approved by the Ethics Committee of the Second Xiangya Hospital of Central South University. The study was reported in line with the ARRIVE guidelines 2.0, with additional supporting documents provided in the supplementary materials.

### Treatment of mice

**Fig. 1:**
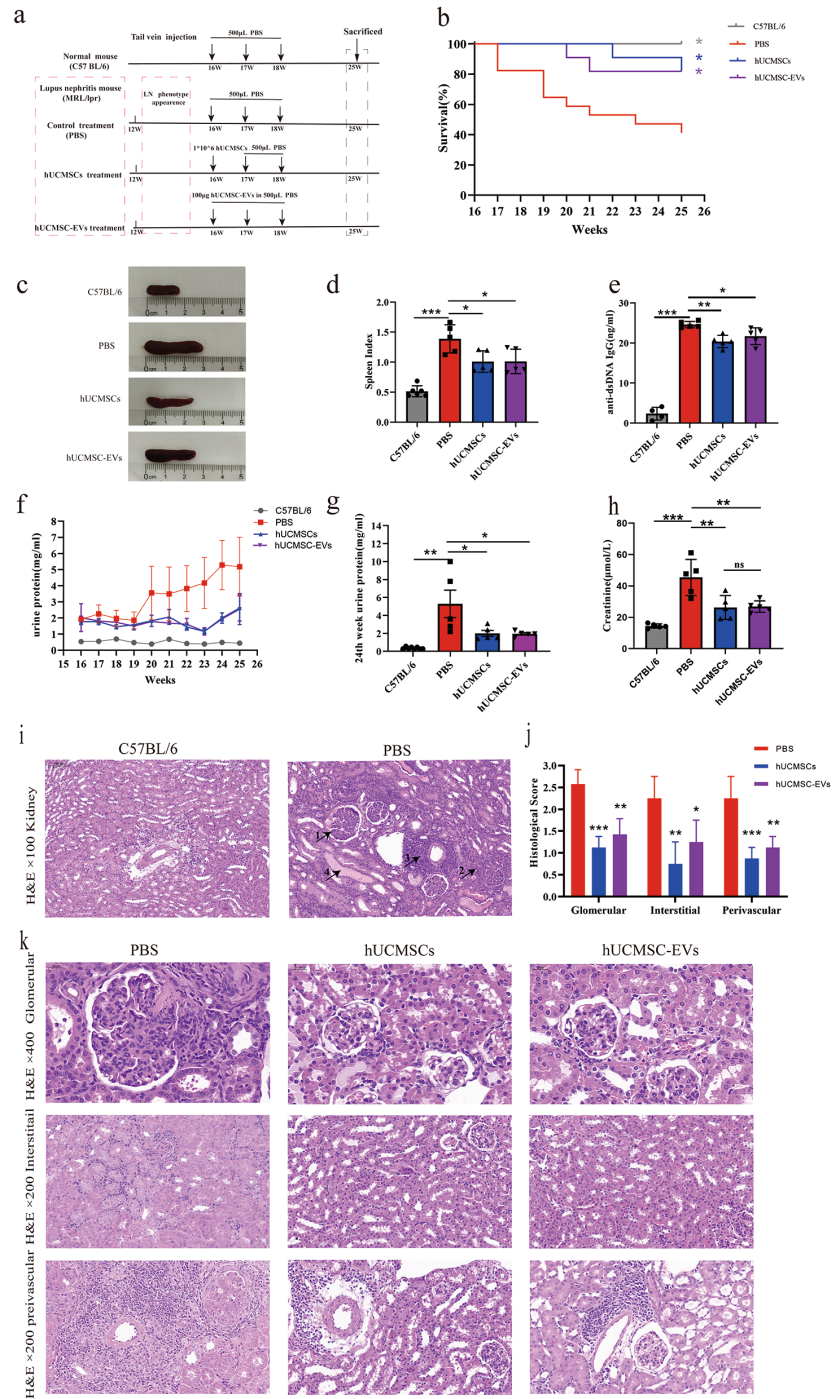
Systemic administration of hUCMSC-EVs ameliorated LN in MRL/lpr mice. (**a**) The schematic diagram for studying the effects of hUCMSC-EVs treatment on experimental LN. (**b**) The cumulative survival rate of each group. The data was combined for two of three independently repeated experiments. The number of animals in the PBS, hUCMSCs, hUCMSC-EVs, and C57BL/6 groups was 17, 11, 11, and 6. (**c**) Spleen morphology and (**d**) spleen index: spleen weight per 100 g of animal body weight, and (**e**) serum anti-dsDNA level were detected at the end of the study. (**f**) Changes in proteinuria level every week during the study. (**g**) The proteinuria level near the end of the study. (**h**) Serum creatinine level was detected at the end of the study. (**i**) Representative kidney sections stained with H&E (original magnification, ×100) in normal mice and MRL/lpr mice treated with PBS: (1) glomerulosclerosis and increased mesangial matrix, (2) interstitial mononuclear cell infiltrations, (3) perivascular mononuclear cell infiltrations, and (4) renal tube cast. (**j**) Histological scores of glomerular, interstitial, and perivascular infiltration of PBS, hUCMSCs, and hUCMSC-EVs treatment of MRL/lpr mice. (**k**) Representative H&E staining of glomerular (original magnification, ×400), interstitial (original magnification, ×200), and perivascular infiltration (original magnification, ×200). Data are expressed as mean ± SD, *p < 0.05, **p < 0.01, *** *p* < 0.001 vs. the PBS group

16-week-old MRL/lpr mice were as models of experimental LN and randomly divided into three groups according to proteinuria level, as follows: phosphate buffered saline (PBS) group (*n* = 12), hUCMSCs group (*n* = 6), and hUCMSC-EVs group (*n* = 6). 16-week-old female C57BL/6 mice were used as normal controls (*n* = 6). To investigate the effect of hUCMSC-EVs on LN, 16-week-old MRL/lpr mice received hUCMSC-EVs at weeks 1, 2, and 3. PBS was used as vehicle control, and hUCMSCs were used as positive controls. All interventions in this study, including PBS, hUCMSCs, and hUCMSC-EVs, were injected via the tail vein of the mice. In week 10, the plasma, kidney, and spleen were harvested from all mice (Fig. 1a). Details of the intervention are shown in Fig. 1a. At the end of the experiments, euthanasia was performed using sodium pentobarbital, following the American Veterinary Medical Association Guidelines for the Euthanasia of Animals (2020 Edition). The experiment was repeated three times independently, and the results presented in this manuscript are mainly derived from the third experiment unless otherwise specified.

### Isolation and identification of hUCMSC-EVs

The expanded hUCMSCs met the minimum criteria for the essential characteristics of MSCs [19] (Supplemental Fig. 1). The hUCMSC-EVs were isolated as described in our previous study [15]. Briefly, P4-P6 hUCMSCs were cultured in MSCs serum-free complete medium (Clin-Biotechnology, China), and culture supernatants were harvested to isolate hUCMSC-EVs by differential ultracentrifugation. The final protein concentrations of EVs preparations were quantified by Bradford Protein Content Assay Kit (Keygen Biotech, China), and particle size distribution and particle number measurement were analyzed by nanoparticle tracking analysis (NTA) (NanoSight NS300, UK). hUCMSC-EVs morphology was observed by transmission electron microscopy (TEM) (FEI Tecnai G2 Spirit, USA). Analysis of CD9(ab236630, Abcam), CD81(ab79559, Abcam), and CD63(ab134045, Abcam) expression on hUCMSC-EVs was performed by using ExoView (NanoView Biosciences, USA).

### Evaluation of renal injury

Spot urine was collected at the same time every week using the “bladder-massage” method. Proteinuria and serum creatinine were measured with Bradford Protein Content Assay Kit (Jiangsu Keygen Biotech, China) and Creatinine Assay Test Kit (Sangon Biotech, China), respectively, according to the manufacturer’s instructions. Kidney tissue samples were fixed in 4% paraformaldehyde for 24 h and embedded in paraffin For renal histopathology assessment. Three μm sections were prepared for hematoxylin and eosin (H&E) staining. We evaluated kidney pathology as previously described [20]. Briefly, glomerular pathology, interstitial pathology, and perivascular cell accumulation were graded according to a scale of 0–3. Kidney slides were evaluated blindly by two investigators, and the final results were confirmed by a renal pathologist.

### Isolation of splenocytes and KIIs

After sacrifice, spleens were removed, and mice were perfused with 30 ml PBS until complete blanching of the liver and kidney occurred. Then, the kidneys were removed. The spleen was gently ground by a syringe plunger and passed through a 70-μm cell sieve to make single-cell suspensions. Erythrocytes in spleen single-cell suspensions were lysed with red blood cell lysis buffer (BD Pharmingen, USA). The Splenocytes were counted and resuspended in PBS for staining and Flow Cytometry analysis. The capsular layer of the kidneys was dissected away. The Kidneys were digested in RPMI 1640 medium (supplemented with 5% FBS) containing 2 mg/ml collagenase IV (BioFroxx, Germany) and 0.1 mg/ml DNaseI (BioFroxx, Germany) at 37 °C for 45 min on a shaker. The completed digested mixture passed through 70-μm and 40-μm cell sieves sequentially and was centrifuged (400 x *g*, 8 min, 4 °C) to obtain cell pellets. Immune cells were isolated by using percoll density-gradient centrifugation [21]. Briefly, the above-mentioned pellet was resuspended with 3 ml of 36% percoll centrifugation medium, then gently transferred to the upper layer of 3 ml of 72% percoll centrifugation medium without any disruption, and centrifuged (1,000 x *g* without brake, 20 min, 4 °C). Immune cells were located at the interface, and the cells were then washed, counted, and resuspended in PBS for staining and Flow Cytometry analysis.

### Flow cytometry analysis

The cells were incubated in PBS. The following antibodies were used in this study. (1) For Th1 (CD4^+^IFN-γ^+^) and Th17 (CD4^+^IL-17 A^+^) cells: zombie NIR, BV750 anti-mouse CD45, BV605 anti-mouse CD3, BB400 anti-mouse CD4, FITC anti-mouse CD8, APC anti-mouse IFN-γ, and BV421 anti-mouse IL-17 A. (2) For DNT (CD3^+^CD4^−^CD8^−^) and Treg (CD4^+^CD25^+^FOXP3^+^) cells: zombie NIR (), BV750 anti-mouse CD45, BV605 anti-mouse CD3, BB400 anti-mouse CD4, FITC anti-mouse CD8, APC anti-mouseCD25, and PE anti-mouse Foxp3. (3) For B cell (CD45^+^CD19^+^CD3^−^), B1 cell (CD19^+^CD5^+^), and plasma cell (CD3^−^CD19^−^CD138^+^): zombie NIR, BV750 anti-mouse CD45, BV605 anti-mouse CD3, PE anti-mouse CD138 and APC anti-mouse CD5. All antibodies were purchased from BD Pharmingen. For Th1 and Th17 cell detection, the cells were stimulated with a leukocyte activation cocktail (BD Pharmingen^TM^550583, USA) at 37 °C for 6–8 h. For intracellular cytokine or intranuclear cytokine staining, the cells were fixed and permeabilized with a Cytofix/Cytoperm Soln kit (BD Pharmingen) or Transcription Factor Buffer Set (BD Pharmingen) according to the manufacturer’s protocol. Data were acquired by flow cytometry (CytekNorthern Lights™, USA) and analyzed with FlowJo software (Tree Star, USA).

### Real-time polymerase chain reaction (RT-PCR)

RNA was extracted from kidney tissues using Trizol (invitrogen, USA). For cDNA synthesis, reverse transcription was performed from 1 μg of total RNA using the Evo M-MLV RT Mix Kit with gDNA Clean for qPCR Ver.2 (Accurate Biology, China). Quantitative real-time PCR assays were performed using the SYBR Green Premix Pro Taq HS Qpcr Kit (Accurate Biology, China) and the StepOnePlus Real-Time PCR Systems (Roche, USA). The relative expression of each gene was determined and normalized to the expression of housekeeping gene glyceraldehyde 3-phosphate dehydrogenase (GAPDH) by using the 2^–ΔΔCt^ method. Gene-specific primers (Tsingke Biotechnology, China) are listed in Supplemental Table 1.

### ELISA

Serum anti–double stranded DNA IgG antibody (anti-dsDNA IgG Ab) was measured by using the mouse anti-dsDNA IgG (Cusabio, China) ELISA Kit. Cytokine levels in the serum were assayed by using mouse IL-2, mouse IFN-γ, mouse IL-17 A, and mouse TGF-β1 ELISA Kit (Elabscience, China) according to the manufacturer’s instructions.

### Western blot analysis

Mice kidney total proteins were extracted by using a mixture of protease inhibitor, phosphatase inhibitor, and Radio Immunoprecipitation Assay (PIPA) lysate buffer (Jiangsu Cowin Biotech, China). Anti-STAT3 (1:1000, ab68153, Abcam), Anti-phosphorylated-STAT3 (1:4000, ab76315, Abcam), Anti-IL-17 A (1:1000, ab302922, Abcam), and Anti-GAPDH (1:6000, ab8245, Abcam) antibodies were used to probe the blots according to standard procedures. Immuno-reactive bands were detected by enhanced chemiluminescence (ECL) technique and visualized using ChemiDoc™ Touch Imaging System (Tanon). The intensity of the blots was quantified by densitometry using Image J software.

### Statistical analysis

All values are expressed as the mean ± standard deviation (SD). We assessed data for normal distribution and similar variance between groups. Statistical analyses were performed by One-way ANOVA for multiple comparisons with Dunnett’s multiple comparisons test on posttests. For survival analysis, the log-rank test was used to compare survival curves. We used GraphPad Prism version 8.3 (GraphPad Software, CA) for statistical analysis. *P* value < 0.05 was considered to be statistically significant.

## Results

### Characterization of hUCMSC-EVs

**Fig. 2:**
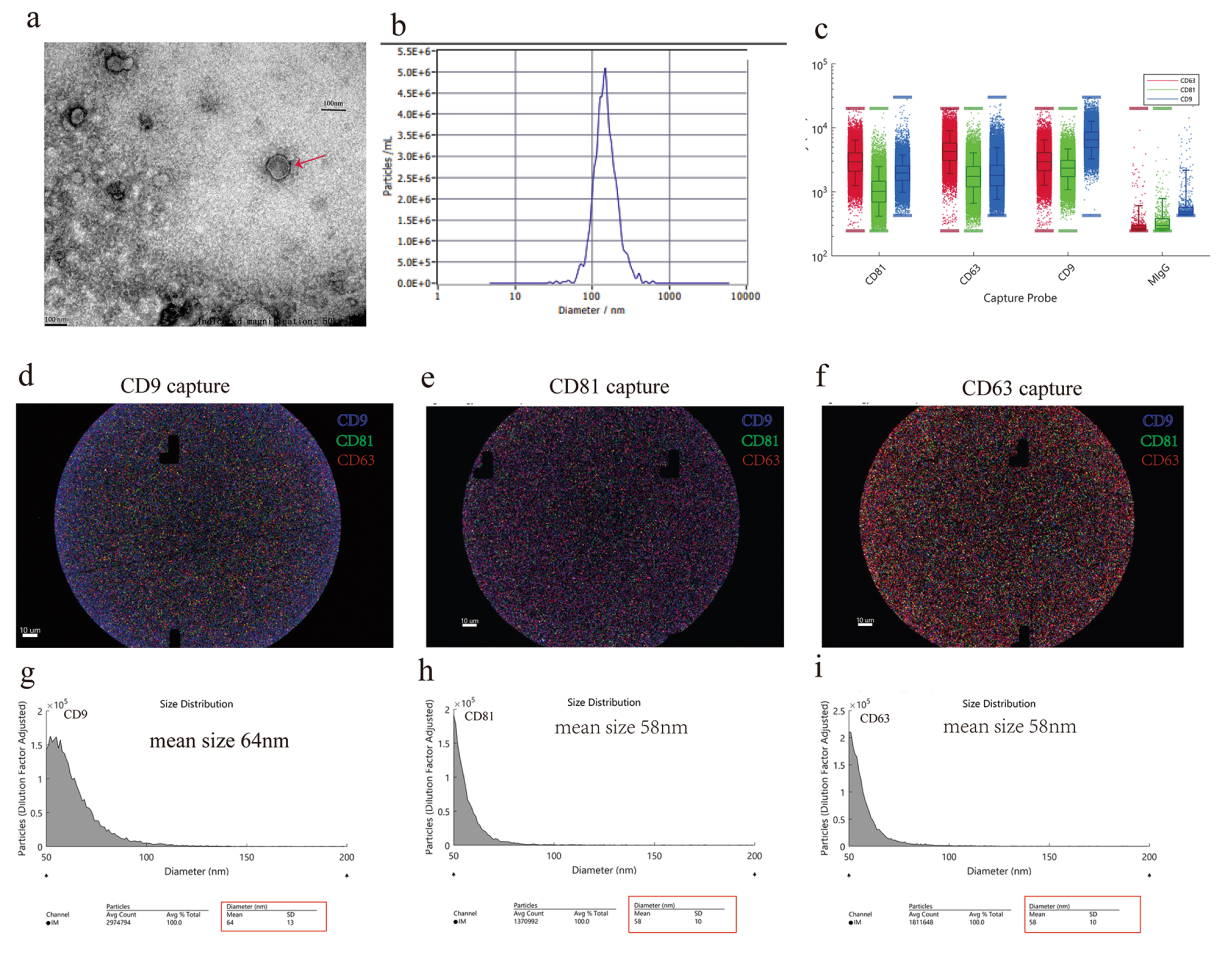
Characterization of hUCMSC-EVs. (**a**) Representative results of the nano-size vesicles photographed by transmission electron microscope. (**b**) Representative results of nanoparticle tracking analyses of the hUCMSC-EVs (1:4000 dilution with particle-free PBS). ExoView analysis of tetraspanin presentation on single EV: (**c**) The fluorescence box plot of CD9, CD81, and CD63; (d–f) Unlabeled EVs were captured on CD9, CD63, or CD81 antibody chips, and EVs were stained with anti-CD9 (blue), anti-CD81 (green), and anti-CD63 (red); (g–i) Size distribution of hUCMSC-EVs captured on CD9, CD63, or CD81 antibody chips

hUCMSC-EVs were observed by TEM with a complete characteristic lipid bilayer (Fig. 2a). NTA showed that the particle size distribution of the hUCMSC-EVs was a single peak with an average particle size of 146.9 nm (Fig. 2b). Next, we utilized ExoView analysis from a single EV level to determine the markers of hUCMSC-EVs. It showed that hUCMSC-EVs simultaneously expressed transmembrane proteins CD9, CD63, and CD81 (Fig. 2d–f), and the expression of CD9 was the highest (Fig. 2c). In addition, the mean particle sizes of hUCMSC-EVs captured by CD9, CD81, and CD63 antibodies were about 64 nm (Fig. 2g), 58 nm (Fig. 2h), and 58 nm (Fig. 2i), respectively. In this study, we found that the yield of 1 × 10^6^ hUCMSCs was about 5.18 μg, and the particle/protein ratio was about 0.6 × 10^9^ particles/μg.

### Systemic administration of hUCMSC-EVs ameliorated lupus nephritis in MRL/lpr mice

Intravenous hUCMSC-EVs improved the survival rate of MRL/lpr mice. The survival rate in the PBS group was 41%, while the hUCMSC-EVs group could be significantly increased to 82% (Fig. 1b). hUCMSC-EVs treatment alleviated lymphoproliferation and decreased autoantibody levels. The spleen index was positively correlated with lymphocyte proliferation, and hUCMSC-EVs treatment significantly reduced the spleen index of MRL/lpr mice (Fig. 1c-d) and reduced serum anti-dsDNA levels (Fig. 1e). hUCMSC-EVs treatment improved renal functions. In general, the extent of proteinuria indicates the severity of nephritis in MRL/lpr mice; the results showed that proteinuria levels in MRL/lpr mice increased progressively with ageing compared with normal mice (Fig. 1f). hUCMSC-EVs treatment delayed the increase in proteinuria (Fig. 1f) and reduced the extent of proteinuria (Fig. 1g), as well as decreased the levels of serum creatinine (Fig. 1h). hUCMSC-EVs treatment attenuated renal damage. Results of H&E staining demonstrate that MRL/lpr mice (PBS group) exhibited severe renal damage characterized by glomerulosclerosis, increased mesangial matrix, renal tube cast, and diffuse perivascular and interstitial mononuclear cell infiltrations (Fig. 1i). Histological scoring for glomerulonephritis, interstitial nephritis, and vasculitis of tissue samples confirmed the significant effects of hUCMSC-EVs in protecting the kidneys (Fig. 1j and k). Moreover, hUCMSC-EVs treatment down-regulated immune cell infiltration in the renal interstitium (Fig. 1k).

**hUCMSC-EVs regulated T cell subsets proportion toward an increase of anti-inflammatory effects**in vivo.

**Fig. 3:**
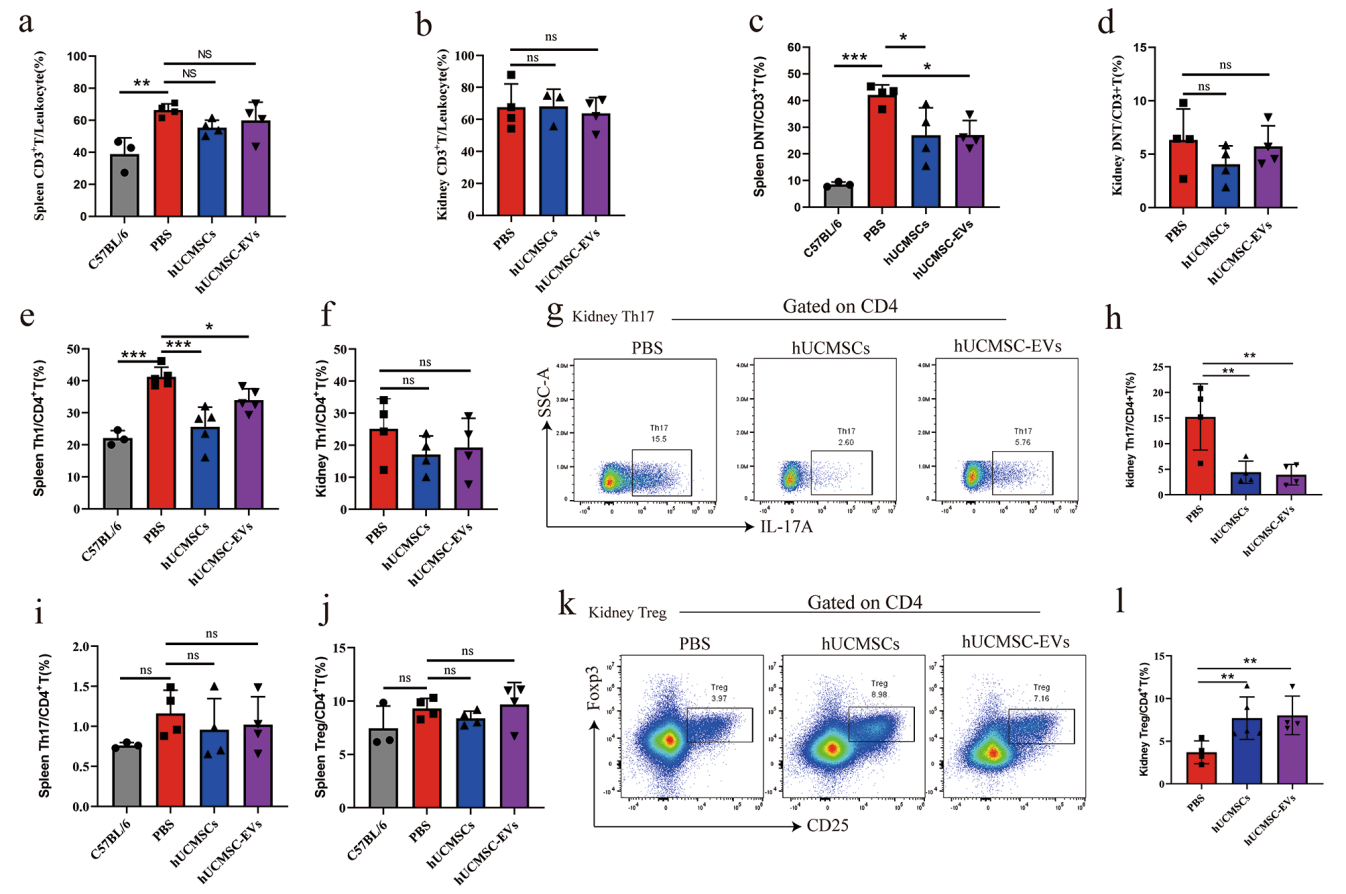
Flow cytometry analyses of T cells among the splenocytes and KITs of MRL/lpr mice after hUCMSC-EVs treatment. (**a**, **c**, **e**, **i**, **j**) Percentages of CD3^+^ T cell, Th1 cell, Th17 cell, and Treg cell among splenocytes of normal mice or MRL/lpr mice treated by PBS, hUCMSCs, or hUCMSC-EVs. (**b**, **d**, **f**, **h**, **l**) Percentages of CD3^+^ T cells, Th1 cells, Th17 cells, and Treg cells among the KITs of MRL/lpr mice treated by PBS, hUCMSCs, or hUCMSC-EVs. (**g**, **k**) Representative flow cytometry analyses images of CD4^+^IL17^+^ (Th17) cells and CD4^+^CD25^+^Foxp3^+^ (Treg) cells among the KITs. Data are expressed as mean ± SD, *p < 0.05, **p < 0.01, *** *p* < 0.001 vs. the PBS group

In the above-described experiment, we found that hUCMSC-EVs alleviated lymphoproliferation of the spleen and decreased immune cell infiltration in the kidney. Then, splenocytes and KIIs were isolated, and the cell populations were analyzed by flow cytometry to further study the effects of hUCMSC-EVs on T cell subsets, primarily focused on pro-inflammatory (DNT, Th1, and Th17 cell) and anti-inflammatory cells (Treg cell). In MRL/lpr mice, we observed CD3^+^ T cell was the main leukocyte type of splenocytes and KIIs (Fig. 3a and b). DNT cell was the predominant cell type among CD3^+^ T cells among splenocytes (Fig. 3c), but the frequency of DNT cells among KITs was lower (Fig. 3d). Compared to PBS treatment, hUCMSC-EVs treatment significantly reduced the frequency of DNT cells among splenocytes (Fig. 3c); however, it had no significant effect on DNT cells among KITs (Fig. 3d). Given the pathogenic role of CD8 + T cell infiltration in lupus nephritis, we concerned CD8 + T cells of KITs and showed that the frequency of CD8 + T cells to CD3 + T cells did not differ significantly between groups of mice (Supplemental Fig. 2). Next, CD4^+^ T cell subsets, including Th1, Th17, and Treg cells, were analyzed. We observed that the frequency of Th1 cells among splenocytes of MRL/lpr mice was significantly higher than in normal mice (Fig. 3e). In contrast, the frequency of Th17 or Treg cells among splenocytes had no significant difference with normal mice (Fig. 3i and j). Then the activation inhibition status (CTLA4) and proliferation (Ki67) of Treg cells by flow cytometry showed that hUCMSC-EVs had no significant effect on Ki67 and CTLA4 expression in Treg cells of MRL/lpr mice (Supplemental Fig. 3). Compared to PBS treatment, hUCMSC-EVs treatment significantly reduced the frequency of Th1 cells among splenocytes (Fig. 3e), however, it had no significant effect on Th17 or Treg cells among splenocytes (Fig. 3i and j) or Th1 cells among KITs (Fig. 3f). Notably, in MRL/lpr mice, the frequency of Th17 cells among KITs was higher than that among splenocytes (Fig. 3g and h). Moreover, compared to PBS treatment, hUCMSC-EVs treatment significantly reduced the frequency of Th17 cells among KITs (Fig. 3g and h), meanwhile it increased the frequency of Treg cells among KITs (Fig. 3k and l). Altogether, our results demonstrated that hUCMSC-EVs were able to reduce the frequency of Th1 cells and DNT cells in spleen and Th17 cells in kidney, thus it reduced the inflammatory phenotype in T cell subset.

### hUCMSC-EVs regulated B cell subset proportion and reduced the number of plasma cell in vivo

**Fig. 4:**
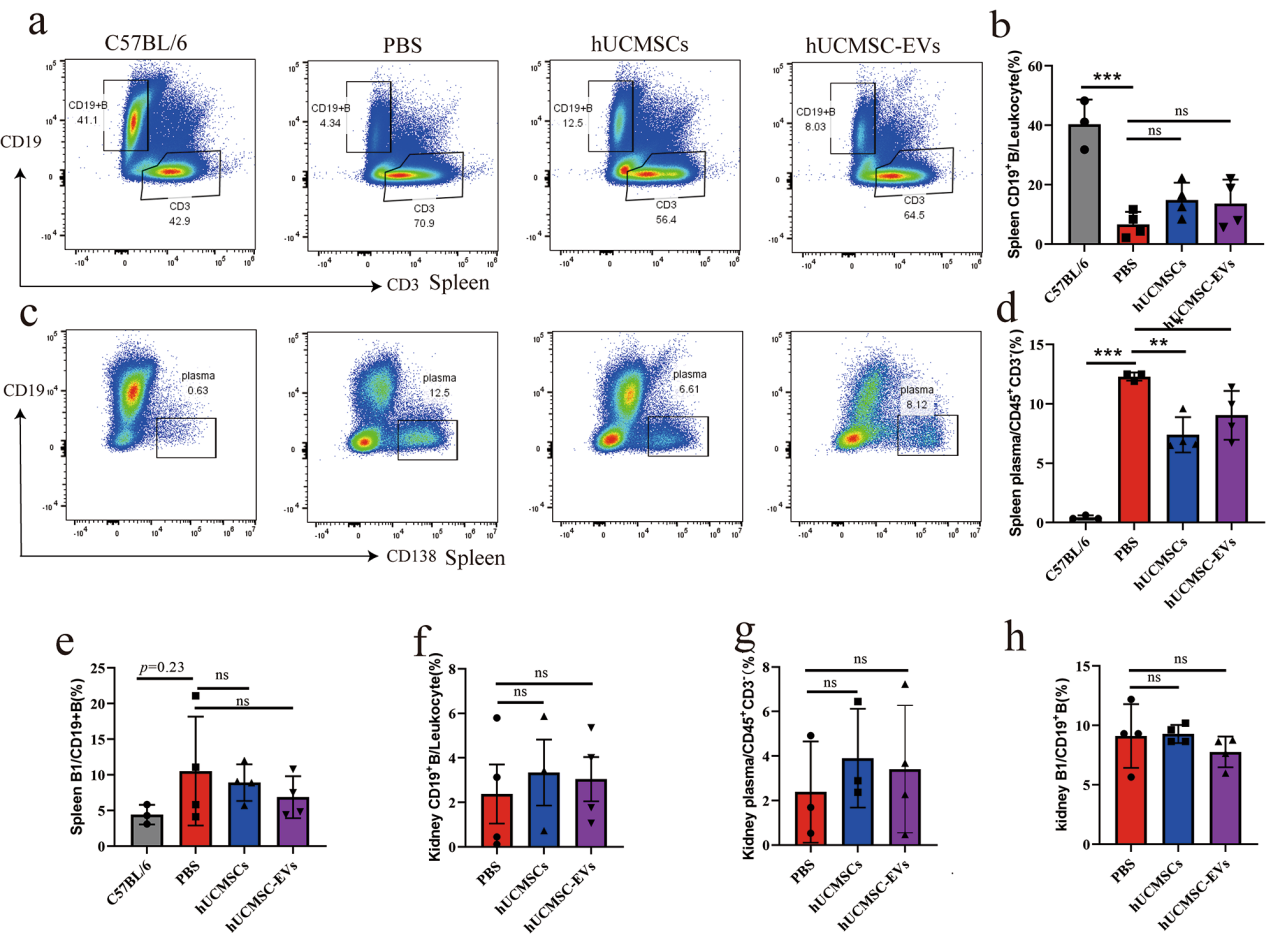
Flow cytometry analyses of B cell, plasma cell, and B1 cell among splenocytes and KIIs of MRL/lpr mice after hUCMSC-EVs treatment. (**a**, **c**) Representative flow cytometry analyses images of CD19^+^CD3^−^ (B cell) and CD45^+^CD3^−^CD19^−^CD138^+^ (plasma cell) among splenocytes. (**b**, **d**, **e**) Percentages of B cell, plasma cell, and B1 cell among splenocytes of normal mice or MRL/lpr mice treated by PBS, hUCMSCs or hUCMSC-EVs. (**f**, **g**, **h**) The frequency of B cell, plasma cell, and B1 cell among KIIs of MRL/lpr mice treated by PBS, hUCMSCs, or hUCMSC-EVs. Data are expressed as mean ± SD, *p < 0.05, **p < 0.01, *** *p* < 0.001 vs. the PBS group

The above-mentioned results showed that hUCMSC-EVs treatment significantly decreased serum anti-dsDNA IgG levels in MRL/lpr mice. Next, we aimed to investigate plasma cells and B1 cells, which are related to autoantibody production. We observed that compared to normal mice, while the CD19^+^ B cell frequency significantly decreased among splenocytes (Fig. 4a and b), the plasma cell frequency significantly increased (Fig. 4c and d). Moreover, there was a trend toward an increased B1 cell frequency among splenocytes of MRL/lpr mice (*p* = 0.23, Fig. 4e). Flow cytometry analysis revealed that hUCMSC-EVs treatment significantly decreased the frequency of plasma cells among splenocytes (Fig. 4c and d), although it had no significant effect on B1 cells among splenocytes (Fig. 4e). In addition, hUCMSC-EVs had no significant effect on B cell, plasma cell, or B1 cell proportion among KIIs (Fig. 4f-h).

### hUCMSC-EVs down-regulated the expression of pro-inflammatory cytokines in vivo

**Fig. 5:**
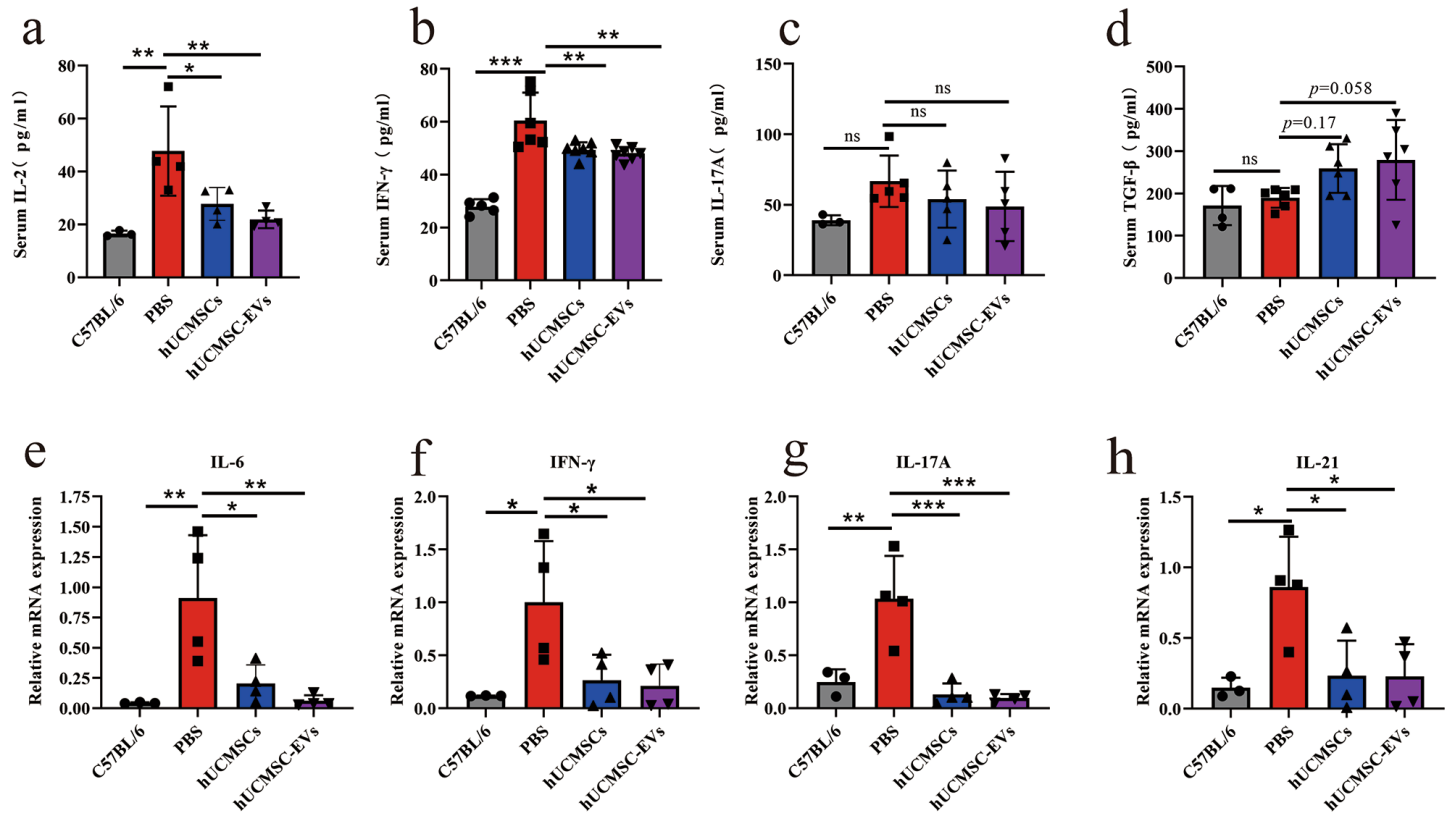
hUCMSC-EVs treatment inhibited the expression of pro-inflammatory cytokines in the serum and kidneys of MRL/lpr mice. (**a**–**d**) The levels of IL-2, IFN-γ, IL-17 A, and TGF-β1 in the serum were detected by ELISA. The data of IFN-γ and TGF-β1 was combined for two of three independently repeated experiments. (**e**-**h**) The mRNA expression of IL-6, IFN-γ, IL-17 A, and IL-21 in the kidney was detected by RT-PCR. Data are expressed as mean ± SD, *p < 0.05, **p < 0.01, *** *p* < 0.001 vs. the PBS group

ELISA analysis revealed that hUCMSC-EVs treatment significantly decreased serum IL-2 (Fig. 5a) and IFN-γ (Fig. 5b) levels in MRL/lpr mice, with a tendency to up-regulate serum TGF-β1 (*p* = 0.058, Fig. 5d) but had no significant effect on serum IL-17 A (Fig. 5c). In addition, real-time PCR analysis revealed that compared to normal mice, the expression of IL-6, IFN-γ, IL-17 A, and IL-21 mRNA was significantly elevated in the kidney of MRL/lpr mice, and hUCMSC-EVs treatment significantly reduced IL-6, IFN-γ, IL-17 A, and IL -21 mRNA expression levels in the kidney (Fig. 5e–h).

### hUCMSC-EVs inhibited the IL-6/ STAT3/ IL-17 a signal pathway in the kidney of MRL/lpr mice

**Fig. 6:**
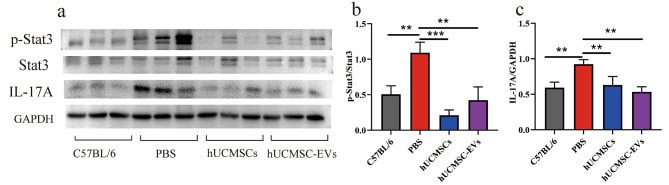
hUCMSC-EVs suppresses the STAT3/Th17/IL-17 A axis in the kidneys of MRL/lpr mice. (**a**) Western blot and (**b**, **c**) quantitative analysis of p-STAT3/STAT3, IL-17 A, and GAPDH protein expression levels. Full-length blots are presented in Supplementary Fig. 4. Data are expres sed as the mean ± SD, *p < 0.05, **p < 0.01, *** *p* < 0.001 vs. the PBS group

In the above-described results, in MRL/lpr mice, we found that hUCMSC -EVs treatment significantly inhibited Th17 cell frequency among KITs and down-regulated the expression level of IL-6 mRNA in the kidney. IL-6 can activate STAT3 and promote the phosphorylation of STAT3 (p-STAT3). Moreover, p-STAT3 plays an important role in regulating the differentiation of naïve CD4^+^ T cells into Th17 cells. The relative expression levels of the phosphorylated Stat3 (p-stat3), Stat3, and IL-17 A in the kidney were evaluated by Western blot analysis to determine the effect of hUCMSC-EVs on the STAT3/Th17/IL-17 A axis. As shown in Fig. 6, the expression ratio of p-Stat3/Stat3 and IL-17 A were significantly up-regulated in the PBS group, which were markedly inhibited by hUCMSC-EVs. Therefore, hUCMSC-EVs inhibited the IL-6/ STAT3/IL-17 A signal pathway in the kidney of MRL/lpr mice.

## Discussion

This study demonstrated that hUCMSC-EVs could alleviate LN in MRL/lpr mice and innovatively focused on their effects on T and B cells among splenocytes and KIIs. We reported, for the first time, that hUCMSC-EVs can treat experimental LN by regulating the Th1/Th17/Treg imbalance and suppressing DNT cells and plasma cells, which might be a novel mechanism for hUCMSCs in the treatment of LN. In addition, this study further revealed that hUCMSC-EVs might attenuate renal injury by inhibiting the IL-6/ Stat3 / IL-17 signal pathway.

MSCs treatment can effectively improve the clinical symptoms of refractory LN patients [22, 23], and its mechanisms involve restoring an immune imbalance and repairing damaged tissues [24, 25]. This study found that hUCMSC-EVs can exert similar therapeutic effects as hUCMSCs, including reducing serum anti dsDNA IgG levels, improving renal function, alleviating renal pathological damage, and increasing the cumulative survival rate of experimental LN mice. It is worth noting that MSC-EVs possess striking advantages over whole-cell therapy, such as low immunogenicity, convenient storage and high biosafety [26]. Therefore, MSC-EVs therapy is expected to be a new and extremely promising strategy for LN.

In SLE, the imbalance in CD4^+^T (known as Th cells) subsets promotes inflammatory responses [4]. Th1 cells are elevated with disease progression in MRL/lpr mice [27], and they promote the LN progression through the overproduction of IFN-γ [28]. Th1 cells in the kidney promote the progression of LN by inducing C3 deposition [20], while hUCMSC-EVs ameliorate renal C3 deposition in MRL/lpr mice [13]. In agreement with these observations, the present study found that hUCMSC-EVs significantly reduced splenic Th1 cells, serum IL-2 and IFN-γ levels and renal IFN-γ mRNA levels. This study demonstrated that hUCMSC-EVs inhibited Th1 cell response.

In SLE patients, the Th17/Treg imbalance has received particular attention, with studies showing an increase in pro-inflammatory Th17 cells [29] and, conversely, a defect in the number or function of Treg cells that suppress inflammatory responses [30]. Clinical evidence has indicated that MSCs exert a therapeutic effect on SLE patients by modulating the Th17/Treg balance [8]. However, in the present study, neither hUCMSCs nor hUCMSC-EVs had a significant effect on the frequency and function of Treg cells among splenocytes of MRL/lpr mice. Studies have reported that the proportion of Th17 cells in the spleen of MRL/lpr mice was not significantly altered during disease progression in MRL/lpr mice [27]. Moreover, in this study, hUCMSCs or hUCMSC-EVs had no significant effect on splenic Th17 frequency or serum IL-17 A, suggesting that hUCMSCs or hUCMSC-EVs might exert therapeutic effects through other mechanisms than the modulation of peripheral Th17 cells or IL-17 A in MRL/lpr mice. The Th17/Treg imbalance in peripheral blood or peripheral immune organs has been well understood in LN patients and experimental LN. However, little is known about it in KITs, presumably because KITs are not easily available. In this study, Th17 and Treg cells were found in KITs of MRL/lpr mice. Compared with the spleen, pro-inflammatory Th17 cell was elevated and anti-inflammatory Treg was decreased in the kidney, suggesting a Th17/Treg imbalance in KITs. The immunomodulatory effects of MSCs on KITs remain unknown, and our results show for the first time that not only hUCMSCs, but also hUCMSC-EVs down-regulated Th17 cells and up-regulated Treg cells in the KITs. This demonstrated that similar to hUCMSCs, hUCMSC-EVs regulated Th17/Treg homeostasis in KITs. Thus, the present study suggested that the organ distribution of Th17 and Treg might differ in LN, and hUCMSC-EVs showed more marked regulation of the Th17/Treg imbalance among KITs than the spleen. This is an interesting discrepancy. A single-cell sequencing study found that the imbalance of infiltrating CD4 + T cells in the kidney is influenced more by the local environment. In contrast, the phenotype of CD8 + T cells may be more similar to that of peripheral cells [31]. This suggested that the mechanism by which hUCMSCs or hUCMSC-EVs reshape the immune homeostasis of KITs may involve inhibiting the inflammatory environment in the kidney. The mechanisms involved remain to be further investigated.

In healthy human peripheral blood, the proportion of DNT(CD3^+^CD4^−^CD8^−^) cells was much lower than that of CD4^+^T and CD8^+^T cells, while it was significantly high to 18–27% in the peripheral blood of SLE patients [32]. DNT cells accumulate in the kidney, and secrete IL-17 [33], and are involved in the pathogenesis of SLE. The reduction in DNT cells in the spleen has been associated with improved survival and delayed progression of glomerulonephritis in MRL/lpr mice [34]. Our study showed that DNT cells are the major immune cells proliferating in the spleen of MRL/lpr mice. Additionally, hUCMSC-EVs significantly reduced splenic hyperplasia and the DNT cell frequency in the spleen. This suggests that hUCMSC-EVs might exert therapeutic effects by inhibiting the proliferation of DNT cells in the spleen.

The IL-6/STAT3/IL-17 signal pathway plays an important role in the differentiation of naïve CD4 + T into Th17 cells. In general, mature APC cells secrete key cytokines (IL-6, IL-21, IL-23, TGF-β, and IL-1β) to activate STAT3, a key transcription factor in naïve CD4^+^T, which drives STAT3 phosphorylation, therefore promoting the differentiation of naïve CD4^+^T to Th17 cells [35]. In addition, renal intrinsic cells (podocytes [36] and mesangial cells [37]) can also play the role of antigen-presenting cells (APCs), which can contribute to Th17 differentiation and activation by presenting eventual danger-associated molecular patterns (DAMPs) to naïve CD4^+^T in the kidney. We found that hUCMSC-EVs inhibited the infiltration of Th17 cells in the kidney. Moreover, our previous study showed that hUCMSC-EVs could regulate Th17 cell differentiation in MRL/lpr mice in vitro [15]. Thus, we demonstrated that hUCMSC-EVs might decrease Th17 differentiation by inhibiting IL-6/STAT3/IL-17 signal pathway. Moreover, Th17 cells and DNT cells in KITs are the main cellular sources of IL-17 A [38] IL-17 A has a criticalt pathogenic role in LN. It is currently believed that IL-17 A bounds to IL-17 receptors on renal intrinsic cells (podocytes, tubular epithelial cells, mesangial cells, and renal endothelial cells), promoting a pro-inflammatory environment, activating fibrotic pathways [38], and leading to structural destruction and impaired function of the kidney [39]. In our studies, hUCMSC-EVs inhibited IL-6/STAT3/IL-17 pathway and alleviated renal pathological injury. Similar to our Studies, the inhibition of IL-6/STAT3/IL-17 pathway play important roles in some disease treatment. In experimental colitis models, histone deacetylase (HDAC) suppressed Th17 differentiation through the IL-6/STAT3/IL-17 pathway, thereby alleviating experimental colitis [40]. Moreover, In immune hepatitis induced by ConA, triptolide may decrease Th17 cells in the liver by regulating IL-6/STAT3-IL-17 signals, thereby exhibiting an immunomodulatory effect and exerting its potential in treating of immune-mediated liver diseases [41]. Taken together, we demonstrated that hUCMSC-EVs inhibited Th17 cell differentiation in KITs by regulating the IL-6/STAT3/IL-17 signal pathway, which might be an important mechanisms of alleviating renal injury.

Aberrant activation of B cells and increased differentiation and survival of plasma cells play a key role in the pathogenesis of SLE [4]. B1 [42] and plasma cells [43] secrete various antibodies including anti-dsDNA, leading to tissue damage. MSCs can inhibit the differentiation of B cells to plasma cells [44, 45], and reduce IgA, IgM, and IgG antibody secretion [44, 45], which are possibly mediated by soluble factors secreted by MSCs [44, 45]. Subsequent studies reported that MSC-EVs [46] could inhibit plasma cells differentiation and antibody production [47]. However, it is unclear whether MSC EVs could affect plasma cells in SLE in vivo, and this study found that hUCMSC EVs reduced plasma cells frequency in spleen and the level of serum anti-dsDNA. It cannot be ignored that current drugs for LN can not inhibit plasma cells well, such as belimumab [48]. This suggests that hUCMSC-EVs inhibited plasma cell might be a promising mechanisms for LN treatment.

B1 cells produce autoantibodies to autoantigens in a T cell-independent manner [42]. In the autoimmune state, B1 cells settled in the abdominal cavity can migrate to the kidney [42]. The elevated B1/B ratio in the kidney of MRL/lpr mice exacerbates the progression of LN [49]. We found that infusion of hUCMSCs and hUCMSC-EVs did not affect the B1/B ratio in the spleen and kidney. Thus, hUCMSC-EVs reduced dsDNA levels probably through other than B1 cells.

This study confirmed the therapeutic potential of hUCMSC-EVs in experimental LN, as well as their modulatory effects on multiple abnormal immune cells, providing a theoretical basis for using of hUCMSC-EVs as cell-free therapies. However, the limitations of this experiment cannot be ignored. We comprehensively investigated the regulatory effects of hUCMSC-EVs on various immune cells, but which cargoes in hUCMSC-EVs exert therapeutic effects and the specific mechanisms remained unknown and need to further investigated.

## Conclusion

The results of this study demonstrated that hUCMSC-EVs had therapeutic effects on experimental LN by regulating Th1/Th17/Treg imbalance as well as inhibiting DNT and plasma cells. Additionally, hUCMSC-EVs inhibited Th17 cell differentiation in KITs by regulating the IL-6/STAT3/IL-17 signal pathway, which might be an important mechanism for alleviating renal injury. Taken together, we demonstrated that hUCMSC-EVs regulating T and B cell immune responses might represent a novel mechanism of hUCMSCs in treating of LN, thus, providing a new strategy for treating LN.

### Electronic supplementary material

Below is the link to the electronic supplementary material.

Supplementary Material 1

Supplementary Material 2

Supplementary Material 3

## Data Availability

All relevant data are included in the manuscript and supplementary material.
