# Supplementary material for: Mesenchymal stem cells-derived extracellular vesicles ameliorate lupus nephritis by regulating T and B cell responses
Source: Stem Cell Res Ther. 2024 Jul 18;15:216. doi: 10.1186/s13287-024-03834-w (PMC11256400; doi:10.1186/s13287-024-03834-w)
Supplement: Supplementary file 2 — Supplementary Material 2 [file 13287_2024_3834_MOESM2_ESM.docx]

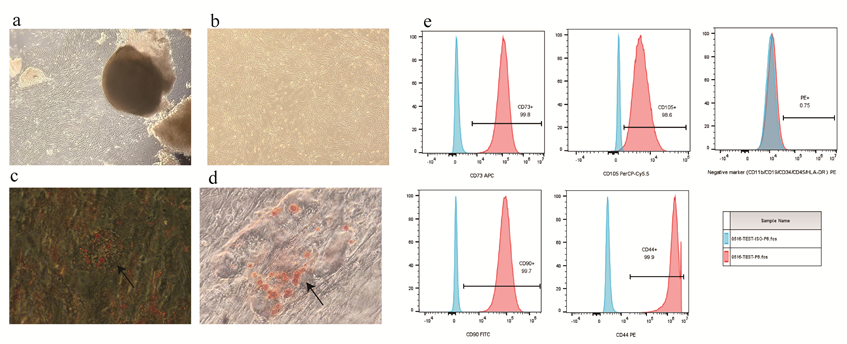


Supplemental Figure1. Characterization of hUCMSCs. (a) The morphological features of hUCMSC were isolated from Wharton’s jelly at day 10 (original magnification, ×40). (b) The morphological features of hUCMSC were cultured in the MSCs serum-free complete medium at passage 3 (original magnification, ×40). (c) Representative images of Oil red O staining of hUCMSCs were cultured under the adipogenic conditions (original magnification, ×400). (d) Representative images of alizarin red staining of hUCMSCs were cultured under the osteogenic conditions (original magnification, ×400). (e) The surface markers of CD73, CD105, CD90, CD44, CD29,CD11b,CD19, CD34,CD45and HLA-DR were analyzed by flow cytometry.


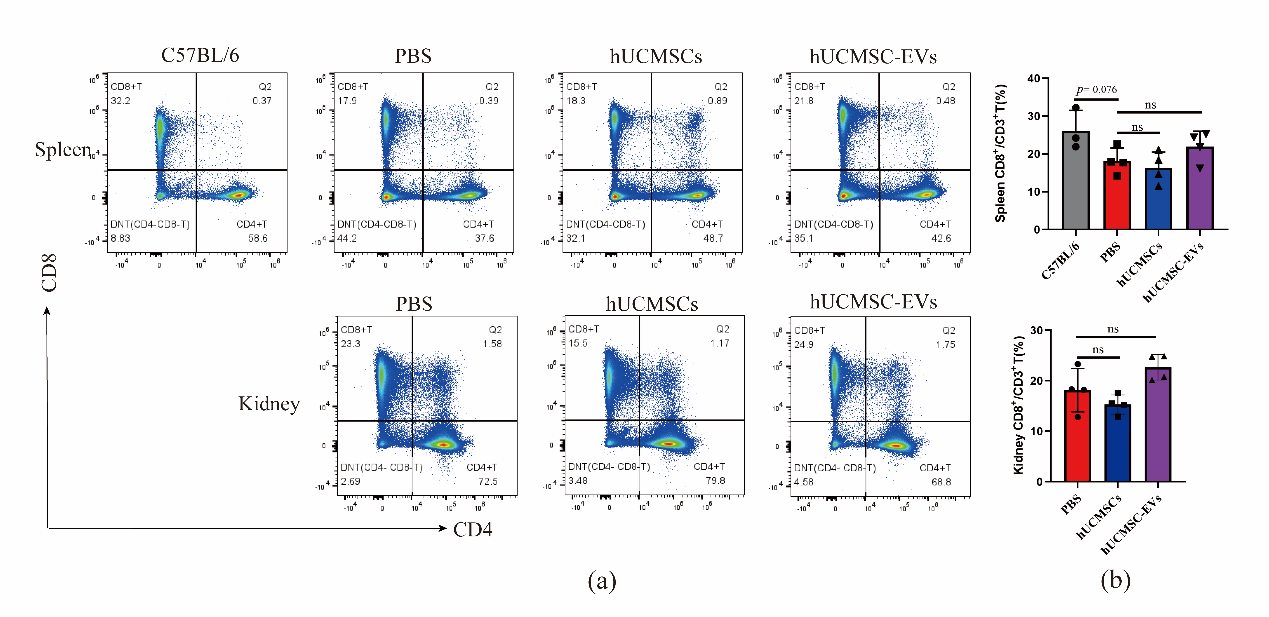


Supplemental Figure 2. Flow cytometry analyses of CD8+ T cells among the splenocytes and KITs of MRL/lpr mice after hUCMSC-EVs treatment. Representative flow cytometry images (a) and statistical graph (b) of CD8+ T cells gated on CD3+ T cells in the spleen (superior) and the kidney (inferior). Data are expressed as mean ± SD.


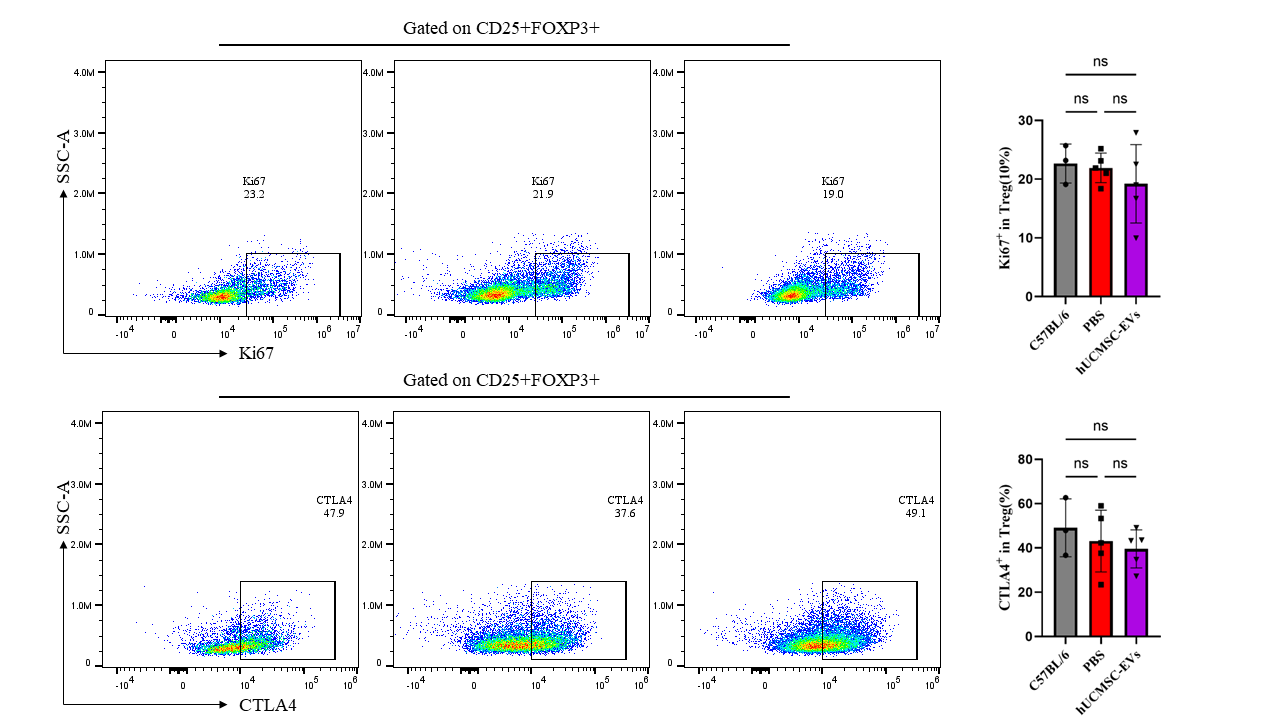


Supplemental Figure 3. Flow cytometry analyses of Treg cells function in the spleen of MRL/lpr mice after hUCMSC-EVs treatment. Representative flow cytometry images and statistical graph of Ki67 (superior) and CTLA4 (inferior). Data are expressed as mean ± SD.

Supplemental Table1 primer sequences

| Primer name | Equence,5’-3’ |
| --- | --- |
| IL-17A | Forward: GCTCCAGAAGGCCCTCAGA |
|  | Reverse: AGCTTTCCCTCCGCATTGA |
| GAPDH | Forward: TGTGTCCGTCGTGGATCTGA |
|  | Reverse: CCTGCTTCACCACCTTCTTGAT |
| IL-6 | Forward: ACTTCCATCCAGTTGCCTTCTTGG |
|  | Reverse: TTAAGCCTCCGACTTGTGAAGTGG |
| IFN-γ | Forward: CAGCAACAGCAAGGCGAAA |
|  | Reverse: CTGGACCTGTGGGTTGTTGAC |
| IL-21 | Forward: GTGAGCATGCAGCTTTTGCC |
|  | Reverse: GGGCCACGAGGTCAATGATG |


