# Supplementary material for: Mesenchymal stem cells-derived extracellular vesicles ameliorate lupus nephritis by regulating T and B cell responses
Source: Stem Cell Res Ther. 2024 Jul 18;15:216. doi: 10.1186/s13287-024-03834-w (PMC11256400; doi:10.1186/s13287-024-03834-w)
Supplement: Supplementary file 3 — Supplementary Material 3 [file 13287_2024_3834_MOESM3_ESM.docx]

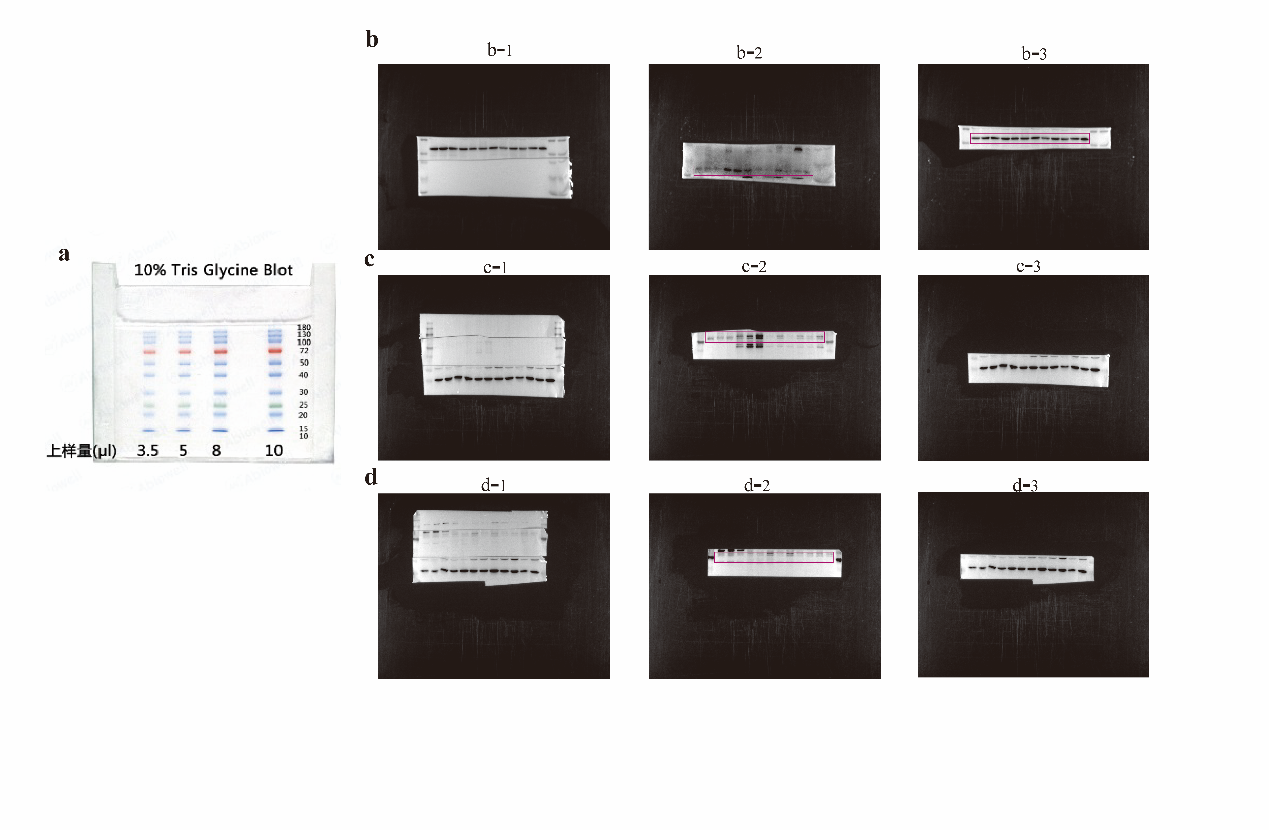


Supplemental Figure 4. Western blot results. **a.** **Pre-stained protein marker band diagram**. **b. IL-17A** b-1, The entire membrane was cut into sections between 25KD-30KD after transfer, then probed with IL-17A and GAPDH antibodies separately, followed by combined detection. b-2, IL-17A band alone was detected, located between pre-stained proteins at 15KD-20KD, approximately 17KD. The red rectangle indicates the cropping position. b-3, GAPDH band alone was detected, located between pre-stained proteins at 30KD-40KD, approximately 36KD. The red rectangle indicates the cropping position. **c. p-STAT3** c-1. The entire membrane was cut into sections between 40KD-50KD and 72-100KD after transfer, then probed with GAPDH, p-STAT3, and p-JAK1 antibodies separately, followed by combined detection. c-2, p-STAT3 band alone was detected, located above pre-stained protein at 72KD, approximately 88KD. The red rectangle indicates the cropping position. c-3, GAPDH band alone was detected, located between pre-stained proteins at 30KD-40KD, approximately 36KD. **d:STAT3** d-1,The entire membrane was cut into sections between 40KD-50KD and 72-100KD after transfer, then probed with STAT3, GAPDH, and JAK1 antibodies separately, followed by combined detection. d-2, STAT3 band alone was detected, located above pre-stained protein at 72KD, approximately 88KD. The red rectangle indicates the cropping position. d-3, GAPDH band alone was detected, located between pre-stained proteins at 30KD-40KD, approximately 36KD.
